# Productivity costs of type 2 diabetes with or without co-occurring substance use disorder and depression

**DOI:** 10.1186/s13561-026-00722-2

**Published:** 2026-01-22

**Authors:** Olli Kurkela, Saara Metso, Leena Forma, Kimmo Suokas, Pekka Rissanen, Jaakko Nevalainen

**Affiliations:** 1 Faculty of Social Sciences, Tampere University, P.O. Box 100, Tampere, 30014 Finland; 2 Department of Public Health, Finnish Institute for Health and Welfare (THL), P.O. Box 30, Helsinki, 00271 Finland; 3 Department of Internal Medicine, Tampere University Hospital, P.O. Box 2000, Tampere, 33520 Finland; 4 Faculty of Medicine and Health Technology, Tampere University, P.O. Box 100, Tampere, 30014 Finland; 5 Faculty of Social Sciences and Business Studies, University of Eastern Finland, P.O. Box 1627, Kuopio, 70211 Finland; 6 Department of Psychology, University of Helsinki, P.O Box 21, 00014 Helsinki, Finland

**Keywords:** Type 2 diabetes, Productivity costs, Friction cost, Human capital, Substance use disorder, Depression, Diabetes comorbidities, Economic aspects

## Abstract

**Background:**

Depression and substance use disorders are common causes of disability and often co-occur with type 2 diabetes (T2D). This study aimed to assess productivity costs using both human capital (HC) and friction cost (FC) methods and to examine how these costs change with age among people with T2D, with or without co-occurring substance use disorders and depression.

**Methods:**

The FinDM database was used to identify individuals of working age (age 30 or older) with T2D (*N* = 377,560) and comorbidities between 1998 and 2017. Individual-level productivity costs were estimated using both the FC and HC methods. The progression of annual mean costs and the associations of T2D and the comorbidities with these costs were analysed using piecewise linear generalized estimating equation models.

**Results:**

People with T2D and the comorbidities incurred over sevenfold annual productivity costs at age 45 compared with those without these comorbidities (€6,320 vs €856). Mean costs showed a notable annual increase (€180 per year) after age 50 years and peaked near the statutory retirement age. At the peak, the annual mean FC and HC estimates were €5,000 and €20,000, respectively. Diagnoses of T2D, substance use disorder, and depression were associated with additional annual FC costs of €53, €195, and €202, respectively.

**Conclusions:**

Co-occurring substance use disorders and depression are associated with higher productivity costs among people with T2D, with substantially stronger associations than those observed for T2D alone. These findings highlight the importance of identifying high-risk individuals and allocating health care resources toward integrated, holistic care.

**Supplementary Information:**

The online version contains supplementary material available at 10.1186/s13561-026-00722-2.

## Introduction

Productivity costs refer to the costs associated with production loss resulting from morbidity and mortality in both paid and unpaid production. In addition to the costs arising from lost production due to absence of a worker, the recruitment and training of a new worker also incur costs. Two common methods to assess productivity costs are human capital (HC) and friction cost (FC) methods. HC and FC methods differ with respect to their perspectives and underlying assumptions, providing insights from organizational and individual standpoints, respectively. In case of permanent exit from the workforce, the HC method measures lost productivity over the expected remaining work life assuming that a person would have been continuously employed and maintained full work ability throughout this period. In contrast, the FC method limits the measurement of lost productivity to a friction period, which represents the time required for the organization to gain back the initial production level. Because estimates obtained using these two methods often differ substantially in magnitude, several economic evaluation guidelines recommend reporting both estimates [1, 2].

Although productivity costs contribute largely to the total burden of diabetes, studies investigating factors that influence them are scarce. In the US, HC estimates of diabetes were estimated to be $90 billion in 2017, constituting 30% of all diabetes-related costs [3]. In Finland, Kurkela et al. (2021) found a notable increase in the risk of early retirement and resulting HC costs after diabetes diagnosis, which were amplified among those with chronic diabetes complications [4]. To date, there has been no assessment of productivity costs of type 2 diabetes using the FC method in Finland.

Heavy alcohol use has been associated with an increased incidence of type 2 diabetes and with the development of acute and chronic diabetes complications, increased healthcare use, morbidity and mortality. Chronic or heavy alcohol consumption impairs insulin action and glucose homeostasis, thereby increasing risk of type 2 diabetes. In addition, relationships between cannabis and opioids and the onset of type 2 diabetes has also been reported, although consistent evidence has not been established [5]. In general, alcohol use disorders can adversely affect work performance, both while at work and through absences [6, 7]. However, evidence on the associations of co-occurring diabetes and substance use disorders on work ability and productivity remains limited.

In Finland in 2017, the age-standardized prevalence of type 2 diabetes was 7%, and 70% of individuals with the disease had onset before age 70 [8]. The prevalence of substance use disorders among working-age individuals with diabetes has not been reported, and the magnitude of the problem remains unclear. Overall, individuals with type 2 diabetes have been shown to consume less alcohol compared to their controls, although a similar reduction has not been observed in alcohol use disorder diagnoses [5]. In the US, type 2 diabetes has also been associated with higher prevalence of illicit drug use (4.2%) compared with the general population (2.1%) [9].

In addition, depression has been associated with a higher incidence of type 2 diabetes, its complications, and poorer treatment outcomes [10, 11]. According to a meta-analysis by Mezuk et al., the pooled relative risk for incident diabetes among individuals with depression was 1.60, while the relative risk for incident depression among individuals with diabetes was 1.15 [10]. This bidirectional association is thought to reflect common biological and behavioral mechanisms: depression may promote diabetes through metabolic and behavioral pathways, while diabetes can aggravate depression through biological stress and disease burden [11, 12].

Globally, approximately 25% of individuals with type 2 diabetes are estimated to have experienced depression [13]. In Finland, the prevalence of depression among individuals with diabetes is not well known. However, a Finnish study from 2008 reported that 26.4% of individuals with previously diagnosed type 2 diabetes had depressive symptoms, compared with 14.4% among those without type 2 diabetes [14]. Depression is also a major cause of disability in Finland, accounting for 31% of the new earnings-based disability pensions granted in 2024 [15].

No previous estimates regarding of the associations between co-occurring type 2 diabetes, substance use disorder, and depression with productivity costs have been reported. This study aimed to assess the productivity costs and their progression with age among people with type 2 diabetes with or without co-occurring substance use disorders and depression. Furthermore, the study aimed to evaluate how the diagnoses of type 2 diabetes and the comorbidities influence the progression of productivity costs. Productivity costs were assessed with both the HC and FC methods. The decision to provide estimates using both methods was based on their different underlying principles and assumptions, and prior evidence showing that they often produce estimates that vary widely in magnitude. The FC method was emphasized because it has seldom been applied to Finnish routinely collected register data and, to our knowledge, never in the context of diabetes in Finland.

## Materials and methods

### Data

The FinDM (Diabetes in Finland) cohort includes all people diagnosed with diabetes in Finland from 1964 to 2017. Data were assembled from several population-wide administrative and healthcare registers using personal identity codes. The dataset included information on diabetes type and diagnosis date, birth and death dates, gender, comorbidity diagnoses and their corresponding dates, occupation, and education. It also contained data on reimbursed long-term sick leaves and pensions. Full details on the database contents, data sources, and cohort formation process are provided by Arffman et al. (2020) [8].

### Study design

This retrospective cohort study included people with type 2 diabetes who were diagnosed in 2017 at the latest, were part of the workforce and aged 30 years or older at least a day between 1998 and 2017. These criteria ensured comprehensive follow-up data for each individual, focusing on the later stages of work life, as socioeconomic status evolves substantially during the first years of adulthood.

Each individual was tracked from either the 1 st of January 1998, or their 30th birthday, until reaching the earliest of the following endpoints: 31 st of December 2017, the commencement of an old age pension, or death. Both fixed-term and permanent early exits from the workforce were identified, as well as the number of days spent outside the workforce each year. Annual productivity costs were then assessed for each individual using both FC and HC methods.

The definition of workforce differed for FC and HC methods. Each year, the FC workforce excluded students, unemployed, and those who were on sick leave or pension for the whole year because these individuals did not directly contribute to paid production in any organization. Conversely, under the HC approach, the workforce included all individuals aged 30 or older until the start of their old age pension or death.

### Productivity costs

The outcome was individual-level productivity costs due to long-term sick leaves (over 9 days), residence- and earnings-based pensions, and premature deaths. Non-market production and lowered productivity while at work (presenteeism) could not be addressed. The occupation and gender-specific annual median wages among the full-time employed Finnish population during the follow-up served as a proxy for the monetary value of the lost productivity for both the FC and HC workforce [16]. The calculation of both FC and HC estimates are described in detail in ESM Text.

The number of days absent was computed as the difference between the end and start days of an absence period. Friction period consists of time required for an organization to place a vacancy, recruit a replacing employee, and provide sufficient training. Time to place a vacancy and complete training was set to 60 days, while the time required to fill a vacancy was estimated using occupation-specific vacancy statistics [17]. In case the departing employee is replaced by an already employed person, a subsequent friction period may be triggered elsewhere, potentially leading to a chain of vacancies. To address this, the initial friction period estimate was adjusted for the length of the vacancy chain (LVC), employing the method described by Targoutzidis et al. (2018) [18].

### Substance use disorder and depression

Individual healthcare histories during the follow-up were searched to identify episodes of substance use disorder (ICD-10 codes F10-F19) or depression (F32, F33) in primary and specialized care. Individuals with at least one such episode were classified as having the comorbidity until the end of the follow-up. All episodes during the follow-up both before and after the diabetes diagnosis were considered.

### Employment status and education

Employment status and education were selected as indicators of socioeconomic status [19, 20]. Employment status affects health through income and various occupation-related factors such as e.g. access to occupational health care, exposure to work environment hazards, and various psychosocial processes. Education, in turn, enhances cognitive functioning, lead to a healthier lifestyle, and facilitate better access and communication with health services.

Employment status was categorized as follows: self-employed, white-collar (upper-level employees and lower-level employees), and blue-collar workers (manual workers). In case of multiple occupations, individual’s most prevalent occupation during the follow-up period was assigned. Education was classified into three levels: primary (primary education), secondary (lower secondary school, upper secondary level, post-secondary non-tertiary), and tertiary (Bachelor’s or equivalent, Master’s or equivalent, Doctoral or equivalent). Each individual’s highest educational level was determined and employed in the analyses.

### Statistical analysis

Descriptive statistics were produced for individual characteristics and annual mean productivity costs by age were computed among both FC and HC workforces. Age group means in productivity costs estimated by the FC method were computed in dynamic subgroups of people (people may shift between subgroups defined by their diagnoses during the follow-up) with different type 2 diabetes and comorbidity status. Furthermore, age group means were investigated by employment status, education level, gender, and birth cohort (1930s and 1940 s, 1950 s and 1960 s, and 1970 s and 1980 s). Regarding analyses with employment status as a factor of interest, the FC workforce was restricted to people with known occupation.

The progression of productivity costs in the FC workforce as a function of age, prior to diabetes diagnosis and from diabetes diagnosis onwards, were examined using piecewise linear models using the generalized estimating equations (GEE) framework, which allows longitudinal investigation and correct inference for dependent data [21]. The progression was assumed piecewise linear, i.e. linear for each decade, with knots placed at 40 and 50 years. In addition, time components were introduced for time since diabetes, substance use disorder, and depression diagnoses; the slopes for these variables thus indicate the extent of acceleration of productivity costs after each diagnosis. Hence, mean costs prior to each diagnosis were estimated on individuals still without the diagnosis, and after the diagnosis, the individual contributed to the estimation of costs after the diagnosis. The model was further adjusted for employment status, education, gender, and birth cohort, which were considered to be potential confounding factors. “Independence” working correlation structure was selected based on the quasi-information criteria. Modelling was based on data available between years 30 and 62 years of age, as preliminary analyses indicated a consistent swift decline in productivity costs after age 62 at latest in all examined subgroups.

Using the model, the progression of costs was illustrated by two scenarios. The scenarios depicted cases where individuals were diagnosed with diabetes at different ages (median and 10th percentile in the FC workforce). In each scenario, the progression of costs was plotted for the following groups: individuals without type 2 diabetes, substance use disorder, and depression, those with type 2 diabetes only, those with type 2 diabetes and substance use disorders, those with type 2 diabetes and depression, and those with all of them. These scenarios aimed to portray a typical case of type 2 diabetes and the examined comorbidities, as well as the impact of earlier onset type 2 diabetes.

Analyses were carried out using R statistical software 4.0.5 [22].

## Results

### Characteristics

The FC workforce comprised 377,560 individuals (Table 1). On average, an individual was followed for 11 years starting at the age of 48 years. Over half of the FC workforce (52%) were born in the 1930 s and 1940 s, contributing to the data at the later stages of their work life and closer to the start of the study period (1998–2017). Within the FC workforce, 65% were diagnosed with type 2 diabetes, 8% with substance use disorder and 10% with depression during their follow-ups. Alcohol use disorder (ICD-10 F10) was the most prevalent substance use disorder diagnosis accounting for 68% of the cases. The median ages at the diagnosis of type 2 diabetes, substance use disorder, and depression did not differ substantially (57, 55, and 53 years, respectively), but the majority of individuals did not develop the comorbidities during the follow-up. White-collar workers constituted 33%, blue-collar workers 28% and self-employed 10% of the FC workforce. A total of 50% had successfully completed secondary level education while 12% had attained a university degree. Men constituted a larger proportion of the FC workforce compared to women (57% vs 43%, respectively). The HC workforce was larger in size (*N* = 425,357) than FC workforce (*n* = 377,560). In other aspects, the two workforces differed only slightly (ESM Table 1).

**Table 1:** Characteristics of the friction cost workforce

N	377,560
Years of follow-up, median; mean (sd)	11; 11 (6)
Age at the start of the follow-up, median; mean (sd)	49; 48 (10)
People with diabetes prior to end of follow-up, n (%)	245,458 (65)
Age at diabetes diagnosis, median; mean (sd)	57; 57 (10)
People with substance use disorder, n (%)	30,207 (8)
Age at first substance use disorder episode, median; mean (sd)^1^	55; 54 (11)
People with depression, n (%)	38,615 (10)
Age at first depression episode, median; mean (sd)^2^	53; 53 (11)
Employment status, n (%)	
Blue collar	107,286 (28)
Self-employed	38,762 (10)
White collar	125,354 (33)
Other^3^	106,158 (28)
Education level	
Primary	144,483 (38)
Secondary	188,503 (50)
Tertiary	44,574 (12)
Gender, n (%)	
Men	216,786 (57)
Women	160,774 (43)
Birth cohort, n (%)	
1930 s and 1940s	194,114 (52)
1950 s and 1960s	163,468 (43)
1970 s and 1980s	19,978 (5)

^1, 2^First event refers to the first event identified during the follow-up and thus, the summary statistics should be interpreted as conditional on experiencing the event

^3^Other category includes people for which employment status was not available in the data

Due to rounding, percentages do not sum up to 100

### Productivity costs

Annual mean productivity costs estimated using both the FC and HC methods exhibited a consistent overall pattern, with costs sharply increasing as the statutory retirement age approached, peaking at age 60 (FC) and 62 (HC), and rapidly declining thereafter until age 65 (ESM Fig. 1).

**Fig. 1:**
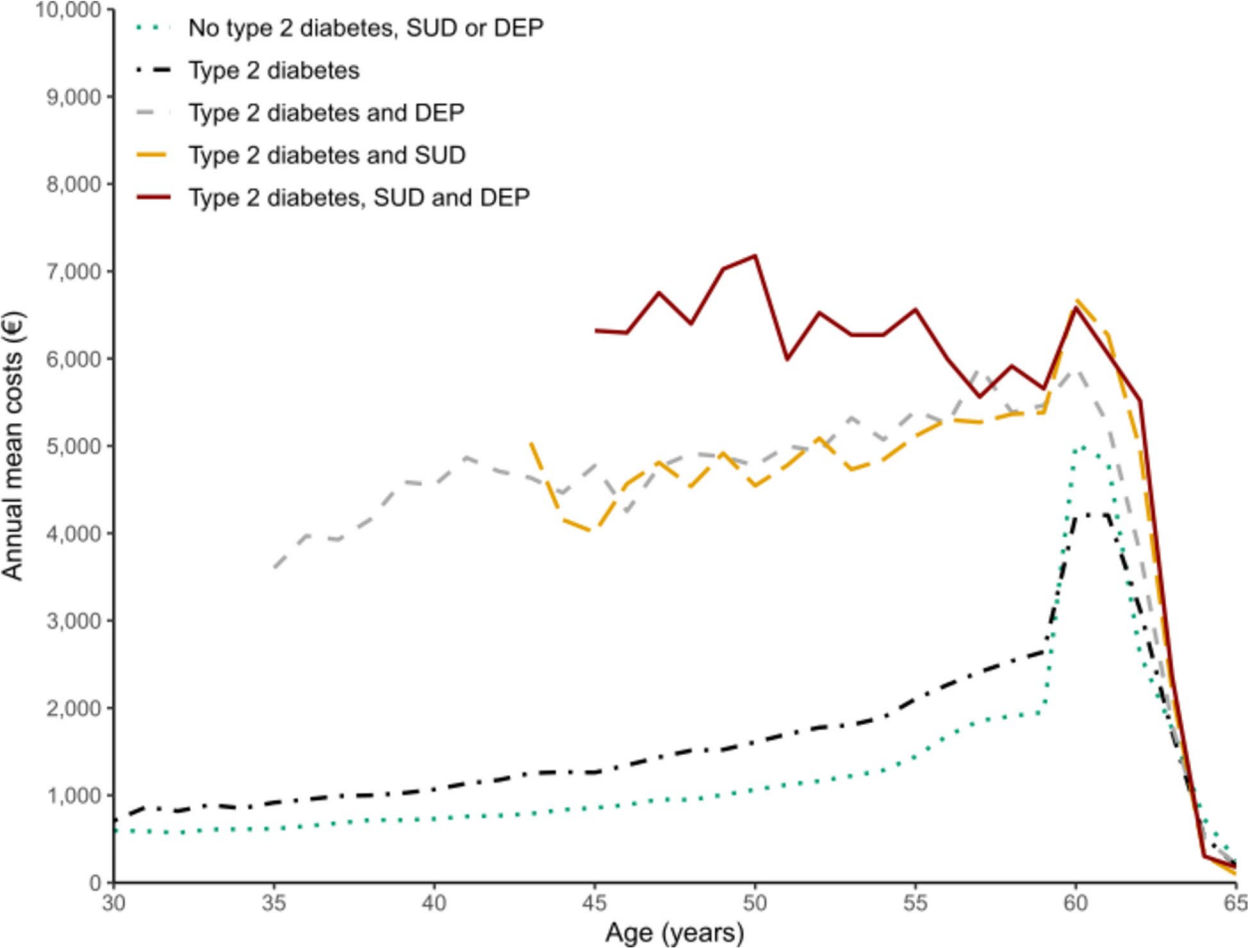
Annual mean productivity costs (€, calculated using the FC method) in the friction cost workforce with and without type 2 diabetes and possibly co-occurring substance use disorder (SUD) and depression (DEP). Only data points based on at least 500 observations are shown

Annual mean productivity costs (FC) of individuals without type 2 diabetes, depression or substance use disorder, those with only type 2 diabetes, those with type 2 diabetes and depression, those with diabetes and substance use disorder, and those with all three diagnoses are shown in Fig. 1. Clear differences in costs were observed between these subgroups across most of the age range, although these differences decreased towards age 60.

The highest mean costs were observed among people with type 2 diabetes, substance use disorder and depression at age 50 (€7,175). At this age the cost differences relative to individuals without these individuals was largest. Individuals with a diagnosed type 2 diabetes exhibited approximately 1.5-fold costs compared to those without type 2 diabetes nearly throughout the entire age range. However, at age 60, the difference reversed (€5,025 vs €4,208).

At age 45, the presence of type 2 diabetes combined with either substance use disorder or depression diagnosis was associated with more than fourfold and fivefold higher costs, respectively, compared with individuals without these diagnoses (€4,010 vs €4,774 vs €856). As age increased toward 60 years, these differences declined substantially.

Blue-collar workers exhibited higher annual mean costs compared to white-collar and self-employed workers across the whole age range (nearly constant difference of €550), escalating to its peak at age 61 (ESM Fig. 2). Individuals with a tertiary level education incurred the lowest annual mean costs across the whole age range when contrasted with those with primary and secondary level educations (nearly constant difference of €530). Gender-based disparities were only evident in the vicinity of the cost peak, with men displaying roughly 20% higher annual mean costs compared to women (€4,997 vs €4,439).

### Impacts of type 2 diabetes, substance use disorders, and depression on productivity costs

According to the longitudinal GEE model evaluating progression (Table 2, Model 1), a one-year increase in age led to an approximate rise of €29 in annual mean costs within the FC workforce. Reaching the age of 40 started a decrease of similar size while reaching the age of 50 initiated over a sixfold increase in annual costs. An additional year with type 2 diabetes, substance use disorder, and depression diagnosis contributed an additional €53, €195, and €202, respectively, to the annual mean costs. The combined impact of the three summed up to €450 increase per year. These impacts changed only to a slight extent after adjusting for employment status, education level, gender and birth cohort (Table 2, Model 2). Interestingly, the impacts of type 2 diabetes, substance use disorder, and depression diagnoses remained consistent with age, which was confirmed with interaction analyses (not reported).

**Table 2:** Results of the longitudinal GEE model for progression of productivity costs in the friction cost workforce, i.e. with the individual-level annual productivity costs (€, calculated using the friction cost method) as the outcome. Only people aged 30 to 62 (the cost peak) were included. In the Model 1, intercept represents the mean of individuals at age 30, who have not been diagnosed with type 2 diabetes, substance use disorder, or depression. In the Model 2, intercept represents the mean of men at age 30, born in the 1950 s or 1960 s, works a blue-collar job, has primary level education, and have not been diagnosed with type 2 diabetes, substance use disorder, or depression

	Model 1	Model 2
N	270,369	270,369
Intercept	572 (571, 573)	952 (951, 953)
Age	29 (29, 30)	39 (39, 39)
Age_40^1^	−35 (−35, −35)	−35 (−35, −35)
Age_50^2^	180 (179, 180)	181 (180,181)
A year with type 2 diabetes diagnosis	53 (53, 53)	57 (57, 57)
A year with substance use disorder diagnosis	195 (194, 195)	183 (183, 183)
A year with depression diagnosis	202 (202, 202)	205 (205,205)
Employment status^3^		
Self-employed		−494 (−495, −493)
White-collar		−547 (−547, −546)
Education^4^		
Secondary		−155 (−156, −155)
Tertiary		−527 (−528, −526)
Women		51 (50, 51)
Birth cohort^5^		
1930 s and 1940s		32 (31, 33)
1970 s and 1980s		196 (195, 197)

^1, 2^The coefficients of variables Age_40 and Age_50 describe the change in slope when reaching age 40 and 50, respectively

^3^Reference is blue-collar workers

^4^Reference is primary level education

^5^Reference is 1950 s and 1960s

**Fig. 2:**
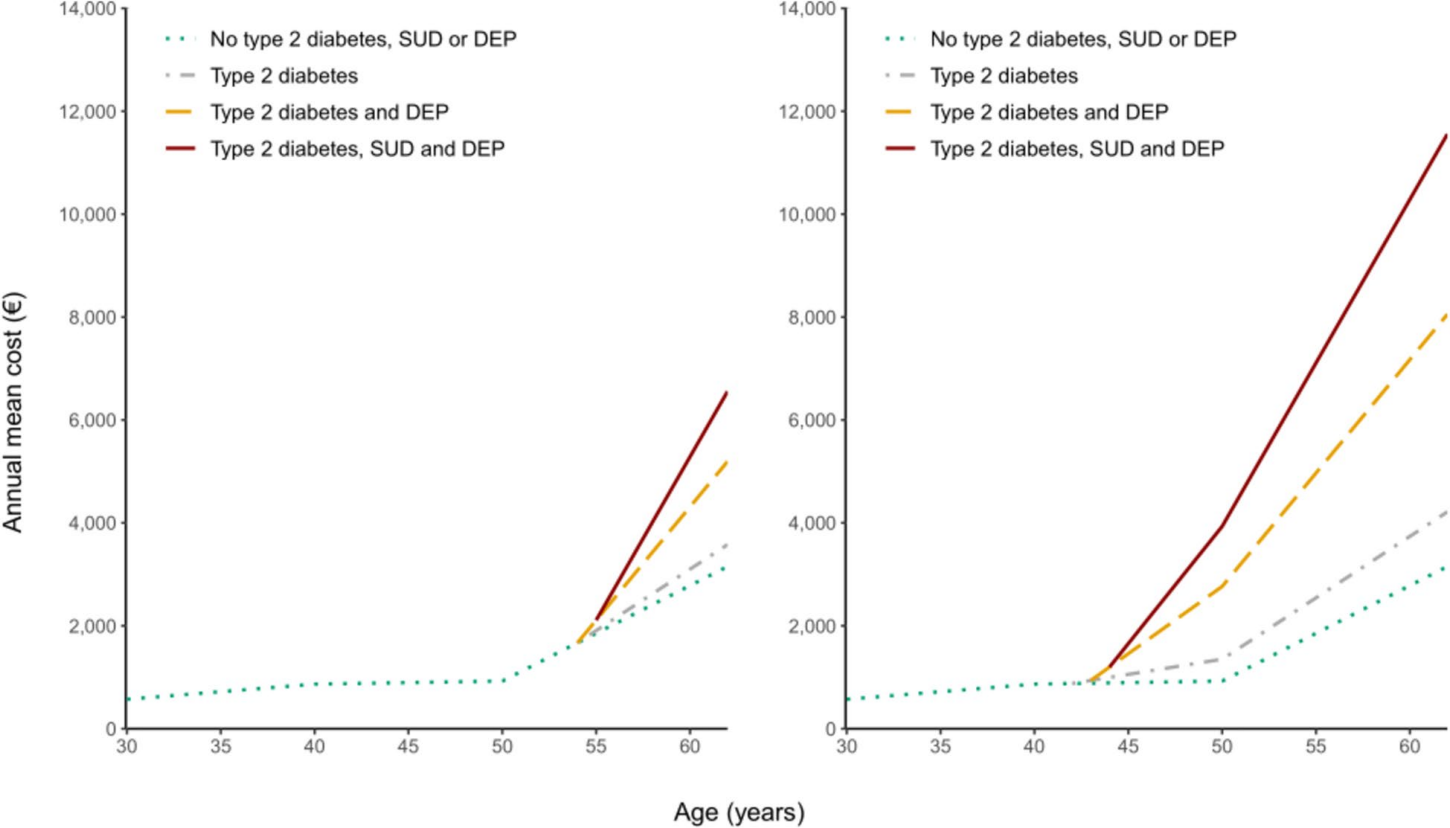
Two scenarios of productivity cost (€, calculated using the FC method) progression before and after type 2 diabetes, substance use disorder, and depression diagnoses estimated by the longitudinal GEE model. In the first scenario (A), type 2 diabetes, substance use disorder, and depression diagnoses are received at ages 54, 55, and 54, respectively. In the second scenario (B), diagnoses take place at age 42, 44 and 43

The median age at type 2 diabetes onset among individuals diagnosed during their work years was 54, while the 10th percentile was 42 years (ESM Table 2). On average, type 2 diabetes, substance use disorder, and depression were diagnosed in close temporal proximity to each other, given that they were diagnosed at all. Based on this information, two scenarios were constructed. In both scenarios, receiving type 2 diabetes, substance use disorder or depression diagnosis initiated a substantial increase in the mean productivity costs. The cumulative impact of co-occurring type 2 diabetes, substance use disorder, and depression was more pronounced in the scenario with type 2 diabetes onset at age 42, resulting in an annual mean of €12,000 at age 62, while in the scenario with the onset of diabetes at age 54, costs reached their peak at €6,000 (Fig. 2).

## Discussion

This study examined the progression of productivity costs resulting from long-term sick leaves, early retirements, and premature deaths among individuals affected by type 2 diabetes and possibly co-occurring substance use disorder and depression, spanning from age 30 to retirement.

We observed a consistent age-related pattern in the productivity costs regardless of the method of estimation. The mean annual productivity costs increased moderately from age 30 to age 40, remained stable until a sharp ascent after age 50, peaked near the statutory retirement age, and then declined rapidly. This trajectory largely aligns with nationally published population-level data, which show a steady increase in disability pensions and reimbursed sick leaves with age up to 50 years, followed by marked increase thereafter. At the peak, the annual mean FC and HC estimates were €5,000 and €20,000, respectively, representing approximately 14% and 55% of the annual median production per person (represented by annual median wage, €36,216) in the full-time working population in 2017.

The productivity costs of individuals diagnosed with type 2 diabetes were approximately 1.5 times higher than among individuals without diabetes across most ages, but this pattern reversed at age 60. A plausible explanation might be the healthy patient effect. The age at diabetes diagnosis correlates negatively with the risk of complications and premature death. Those diagnosed after age 60 may represent individuals who engage with healthcare more actively and have better access through occupational health services, leading to earlier diagnosis of milder, age-associated diabetes that has only limited impact on their work ability and productivity costs [23–25].

Type 2 diabetes alone showed relatively modest productivity costs compared to cases where it co-occurred with substance use disorder or depression. Co-occurrence of substance use disorder or depression with type 2 diabetes was associated with fourfold and fivefold higher productivity costs, respectively, compared to people without the three diagnoses at age 45. Differences decreased towards age 60, when people with type 2 diabetes and both comorbidities demonstrated 1.3-fold higher costs compared to those without, accounting for approximately 18% of the annual median production.

Only 5% and 6% of the disability pensions had diabetes-related diagnoses as primary or secondary cause of disability, respectively, indicating that diabetes itself seldom serves as the primary reason for disability pension. Consistent with this, Virtanen et al. (2015) reported similar findings in the Swedish working age population, showing that disability pensions among people with diabetes are most often granted based on comorbid mental, musculoskeletal, or circulatory diseases [26].

Each of the three diagnoses — type 2 diabetes, substance use disorder, and depression — was associated with an increase in annual mean costs. Importantly, the comorbidity diagnoses were the dominant factors in the development of productivity costs, with each associated with more than threefold increase in annual mean costs compared to type 2 diabetes alone. For example, having a depression diagnosis for three years resulted in a cost increase greater than the disparities between white-collar and blue-collar workers, or between those with primary and tertiary level education.

The co-occurring comorbidities were associated with substantially increased costs, whether diagnosed in the middle (age 42) or later stages of work life (age 54). Consequently, the cumulative effect of living with type 2 diabetes and both comorbidities until age 62 in both scenarios was evident, approximately doubling the overall costs. Earlier-onset type 2 diabetes often exhibit more severe symptoms and higher risk of complications than later-onset cases, making it more challenging to maintain work ability [23, 27]. Chronic diabetes complications have been shown to be associated with an increased risk of early exit from the workforce and related productivity costs. Nevertheless, the majority of individuals with type 2 diabetes are diagnosed closer to the statutory retirement age and therefore do not develop diabetes complications during their working years [4]. Analyses did not adjust for the presence of diabetes complications or lifestyle factors.

The adverse effects of alcohol, drugs, and depression on type 2 diabetes management are well-documented [5, 11, 28]. Alcohol and drugs may also impair self-management and glucose balance in type 2 diabetes, leading to symptoms such as fatigue and anxiety, which can reduce work ability [5, 28]*.* Poor glucose control may further accelerate the development of diabetes-related complications, which are associated with a higher risk of early exit from the workforce [4]. Similarly, Fugunaka et al. (2022) reported an increased risk of long-term sickness absence due to mental health disorders among Japanese individuals with diabetes compared with those without [28].

Both employment status and level of education demonstrated substantial associations with productivity costs. Blue-collar workers incurred higher productivity costs than white-collar workers and self-employed, while individuals with tertiary education had lower average costs than those with secondary and primary level education. A lower socioeconomic status may contribute to these heightened costs, as it has been associated with underutilization of available services due to poorer access to care or challenges in adapting diabetes-related knowledge and adhering to treatments [29]. Consistent with previous research, gender, age, education, and comorbid conditions play significant roles in work disability in addition to diabetes [30]. Virtanen et al. also demonstrated that people with diabetes and mental disorders who were living alone had the highest rates of sickness absence and disability pension days [26].

In the current study, the effect of co-occurring type 2 diabetes, substance use disorder, and depression on productivity costs was reported for the first time in Finland and, to our knowledge, worldwide. These findings are significant, as both substance use and mental health disorders are highly prevalent [31–33]. In Finland, the proportion of individuals who received healthcare services due to a mental health disorder increased by over 50% from 2018 to 2022 [34–36]. This upward trend was evident across all education levels, with a greater increase among those with the higher education and among women compared with men.

This study provided estimates of productivity costs using both FC and HC methods. It primarily focused on the organizational perspective of productivity loss captured by the FC method, which was applied for the first time in Finland in the context of type 2 diabetes. The magnitude of productivity cost estimates strongly depended on the chosen approach: the HC method produced estimates nearly ten times higher than the FC method at the cost peak. Such large differences in magnitude are consistent with the previous literature and arise from the underlying assumptions and parameterization of the methods [37]. The FC method has faced criticism for potentially underestimating the costs due to the chaining of vacancies, whereas the HC method has been scrutinized for overestimating the costs stemming from the assumption of full employment [18, 38]. In this study, the former limitation was addressed by employing the LVC multiplier, resulting in a substantial increase in productivity costs.

In addition, labour market characteristics, such as the unemployment rate, job mobility, and occupational health care coverage, are likely to affect productivity cost estimates, particularly under the FC method. However, these factors are at least partly captured through the operationalization of the friction period, which is based on occupation-specific national statistics on vacancy duration. Finland has below-average job mobility compared with other member countries of the Organisation for Co-operation and Development (OECD), which may contribute to longer friction periods, as well as higher-than-average unemployment rate, which may in turn shorten them [39, 40]. In addition, approximately 90% of Finnish working employees are covered by occupational health care [41]. Regarding the HC method, labour market characteristics related to mobility and replacement are less relevant, and factors other than occupational health care coverage in maintaining work ability of individuals with type 2 diabetes are likely to have only a limited role. Differences between various proxies for lost productivity (the wages with varying specificity) were negligible compared with the choice of method (results not shown). Overall, these findings emphasize the importance for decision-makers to understand and account for methodological differences when interpreting cost estimates.

This study supports the previous finding that Finnish information systems provide a reliable basis for routine monitoring of the productivity loss component of disease burden, which broadens the perspective beyond healthcare and supports more comprehensive decision-making [42]. Nevertheless, collecting country-specific data on additional costing factors, such as compensation mechanisms and multiplier effects, would potentially result in more precise assessments.

The productivity cost estimates in this study are country-specific, and comparisons with other countries with different healthcare, social security and labour market arrangements should be made with caution. In addition, a country’s demographic and socioeconomic structure, overall population health, and cultural patterns in health-seeking behaviour may influence both the magnitude of the estimates and strength of observed associations. From a methodological perspective, producing reliable productivity cost estimates requires access to relevant data, which in turn depends on country’s capacity to collect and maintain such information.

From a policy standpoint, the findings emphasize the need to monitor mental health and substance use in diabetes management, ensure timely support measures, and consider socioeconomic differences in both clinical practice and care policies. Individuals, when diagnosed with one of the examined conditions, should be routinely screened for others, and when necessary, directed to appropriate care to preserve their work ability. This is especially relevant among people receiving diagnosis at an earlier age, as the cumulative effect was shown to be substantial.

There is an ongoing debate about patient segmentation and tailored strategies in diabetes care to allocate limited healthcare resources to those with the greatest needs. A major contribution of this study was the identification of high-risk individuals for whom multidisciplinary treatment strategies to sustain work ability could be particularly beneficial. In a previous Finnish study, people with diabetes remained in the workforce 2.5 years longer compared to population controls suggesting a need to shift the focus to those most at risk of disability [43]. While interactions between type 2 diabetes, the examined comorbidities, and socioeconomic factors were beyond the scope of this study, they represent an important area for further research. Understanding these interactions could further inform the customization of treatment strategies for complex, high-risk groups.

High-risk groups could benefit from early identification of substance use disorders, depression, and anxiety during routine diabetes follow-ups, followed by integrated care that links diabetes management with mental health and substance use services. Workplace interventions, such as flexible schedules and occupational health support, could further help maintain work ability among individuals with diabetes and these comorbidities. Furthermore, socioeconomic inequalities could be addressed by screening for social and economic factors associated with poor self-management or work disability and by offering coordinated support from social workers and occupational therapists [44].

The main strength of this study lies in the use of extensive and representative data encompassing the entire Finnish working-age population with type 2 diabetes. The data allowed for longer follow-up periods than in previous studies, including episodes both before and after type 2 diabetes diagnosis. In addition, data allowed for adjusting for key confounders, including employment status and education, indicating their relevance to the productivity cost estimates.

In the study, early exits from the workforce were not limited to sickness or disability, and diagnosis-specific exits were not in focus. This approach was based on the assumption that morbidity may elevate the risk of all types of exits, in addition to those resulting from disability alone. However, other co-occurring comorbidities, such as musculoskeletal disorders or cardiovascular diseases, may have confounded the estimates.

In addition, employment status information was not available for a substantial proportion of study participants, which may introduce bias in subgroup analyses. As the reasons for missing employment status could not be ascertained, assessment of the underlying missingness mechanism was not possible.

International Classification of Primary Care (2nd edition, ICPC-2) codes commonly used in primary care were not considered, which may have resulted in the omission of certain comorbidity-related healthcare episodes. Combined with the absence of data from most occupational health care, this may have led to the underestimation of comorbidity prevalence.

Furthermore, subjects were classified as having a comorbidity from the first diagnostic episode onward until the end of their follow-up. Because substance use disorders and depression are typically episodic, this classification may have led to exposure misclassification by overstating the duration of exposure and potentially understating the mean costs among individuals with these comorbidities.

In Finland, national registers have only recently begun to include information on short-term sick leaves of less than nine days, and the impacts of diabetes and comorbidities on these absences therefore remain unexplored. Consequently, the productivity costs reported in this study are likely conservative.

To conclude, this study addressed several under-researched aspects of the major contributors to disease burden in Finland and globally. The findings indicate that co-occurring substance use disorders and depression substantially amplify productivity losses among people with type 2 diabetes. A considerable share of these losses could potentially be prevented through a more targeted focus on high-risk groups in diabetes care, such as people with type 2 diabetes and co-occurring substance use disorder and depression, and by sustaining work ability among those affected.

## Supplementary Information

Supplementary Material 1.

## Data Availability

No datasets were generated or analysed during the current study.

## Abbreviations

DEP: Depression
FC: Friction cost
FinDM: Diabetes in Finland
HC: Human capital
ICPC-2: International Classification of Primary Care, 2nd edition
LVC: Length of the vacancy chain
OECD: Organisation for Economic Co-operation and Development
SUD: Substance use disorder
T2D: Type 2 diabetes
