# Supplementary material for: Productivity costs of type 2 diabetes with or without co-occurring substance use disorder and depression
Source: Health Econ Rev. 2026 Jan 22;16:15. doi: 10.1186/s13561-026-00722-2 (PMC12908351; doi:10.1186/s13561-026-00722-2)

## Supplementary materials

### ESM text

### Assessing productivity costs

In Finland, permanent residents are allowed social security benefits, including sick leave reimbursements by Social Insurance Institution (SII) and employment-related benefits by several public and private insurers (35,36). Regarding sick leaves, the employer is responsible for compensating the employee with a full wage for 10 first weekdays of the absence. Sick leaves exceeding 10 days are reimbursed by the SII (35). Data on both are routinely collected in registers administered by SII and the Finnish Center for Pensions, however data do not cover sick leaves that are under employers’ deductible period.

The outcome of this study was individual-level productivity costs. Productivity costs were restricted to those arising from loss of paid production due to absences resulting from sickness, disability, and deaths. Non-market production such as informal care, volunteer work, and lowered productivity while at work (presenteeism) could not be addressed. The cost components included lost productivity resulting from long-term sick leaves, residence- and earnings-based pensions, and premature deaths, regardless of their type or whether or not they were granted based on diabetes or the examined comorbidities. To successfully apply both the FC and HC methods, it is crucial to reliably estimate 1) the frequency and lengths of work absences and friction periods and 2) the value of lost productivity in an appropriate way. (37)

#### Length of the absence

The number of days absent was computed as the difference between the end and start days of an absence period. However, data regarding the end date of a pension was only available for fixed-term pensions. Consequently, an assumption was made that all indefinite pensions lasted until the conclusion of an individual’s follow-up, or until they were substituted by another pension or death. In case of premature death, the end of the absence period was determined as the annual average retirement age (63 throughout the study period). As the reimbursement of sick leaves only start after ten days in Finland, 10 days were added to each sick leave period. Acknowledging that part-time pensions (12% of all earnings-based pensions) entail only partial productivity reduction, a multiplier of 0.475 was employed. This multiplier reflected the common range of 35-70% of regular earnings for individuals under part-time pension status (38).

#### Friction period

With the FC method, the lost productivity was constrained to a friction period. This period refers to the time required for an organization to place a vacancy, recruit a replacing employee, and provide sufficient training. The time required for an employer to place a vacancy and train a new employee was assumed to be 60 days, consistently with the previous literature (34). The time required to fill a vacancy was estimated using occupation-specific vacancy statistics (15).

In case the departing employee is replaced by an already employed person, a subsequent friction period may be triggered elsewhere, potentially leading to a chain of vacancies. To address this, the initial friction period estimate was adjusted for the length of the vacancy chain (LVC) employing the method introduced by Targoutzidis et al. (2018) (16). In the proposed adjustment, the initial friction cost estimate was multiplied by the product of two factors: the ratio of already employed job seekers and all job seekers in the population, and the odds of being employed. The requisite data for these parameters were acquired from the publicly available database. (15)

Further approaches to potentially improve the accuracy of estimates, such as accounting for compensation mechanisms and multiplier effects, have been proposed in the literature (39). These were not considered in the analysis, as data to evaluate the required parameters within the context of Finland was not available.

#### Valuation of lost productivity

The annual occupation and sex-specific median wages among full-time employed population served as a proxy for the monetary value of the lost productivity for both FC and HC methods. Estimates were sourced from the publicly available database of Statistics Finland and subsequently adjusted to reflect the 2017 wage level using the index of wage and salary earnings (40). In case the information on occupation was not available for an individual, sex-specific median wage was used instead.

To calculate the productivity cost estimate, the annual wage estimate was multiplied by each individual’s proportion of days absent per year (365). With the FC method, in case the actual absence period was shorter than the estimated friction period, the actual absence period was used. Vice versa, if the length of the actual absence period exceeded the friction period, the friction period was utilized instead.

### Supplementary Tables

**ESM Table 1.** Characteristics of the human capital workforce.

|  |  |
| --- | --- |
| N | 425,357 |
| Years of follow-up, median; mean (sd) | 14; 13 (6) |
| Age at the start of the follow-up, median; mean (sd) | 50; 49 (10) |
| People with diabetes prior to end of follow-up | 307,558 (72) |
| Age at diabetes diagnosis, median; mean (sd) | 58; 57 (11) |
| People with substance use disorder, n (%) | 35,004 (8) |
| Age at first substance use disorder episode, median; mean (sd)^1^ | 53; 52 (10) |
| People with depression, n (%) | 46,975 (11) |
| Age at first depression episode, median; mean (sd)^2^ | 52; 51 (11) |
| Employment status, n (%) |  |
| Blue collar | 109,058 (26) |
| Self-employed | 38,980 (9) |
| White collar | 126,331 (30) |
| Other^3^ | 150,988 (35) |
| Education level |  |
| Primary | 174,742 (41) |
| Secondary | 204,346 (48) |
| Tertiary | 46,269 (11) |
| Gender, n (%) |  |
| Men | 244,240 (57) |
| Women | 181,117 (43) |
| Birth cohort, n (%) |  |
| 1930s and 1940s | 235,580 (55) |
| 1950s and 1960s | 168,084 (40) |
| 1970s and 1980s | 21,693 (5) |

^1, 2^ First event refers to the first event identified during the follow-up.

^3^ Other category includes people for which employment status was not available in the data.

Due to rounding, percentages do not sum up to 100.

**ESM Table 2.** Statistics on difference in diagnosis times of type 2 diabetes and examined comorbidities. Age range 52-56 and 40-44 includes the median and 10^th^ percentile of age at diabetes diagnosis within the FC workforce who had diabetes diagnosed after age 30 and prior to retirement.

|  | People with diabetes diagnosis at 52-56 with a comorbidity diagnosis | | People with diabetes diagnosis at 40-44 with a comorbidity diagnosis | |
| --- | --- | --- | --- | --- |
|  | Time from T2D to SUD | Time from T2D to DEP | Time from T2D to SUD | Time from T2D to DEP |
| N | 5730 | 7920 | 1556 | 2210 |
| 10th percentile | -11 | -11 | -9 | -9 |
| Median | 1 | 0 | 2 | 1 |
| 90th percentile | 12 | 11 | 16 | 15 |

### Supplementary Figures

**ESM Figure 1.** Annual mean days absent (A) and productivity costs (B) in the friction cost and human capital workforces. FC (base) stands for productivity costs calculated using friction cost method. FC (adjusted) stands for friction cost estimates adjusted for length of vacancy chain. HC stands for human capital estimates. In each case, the absent days were valued using sex- and occupation specific annual median wages.


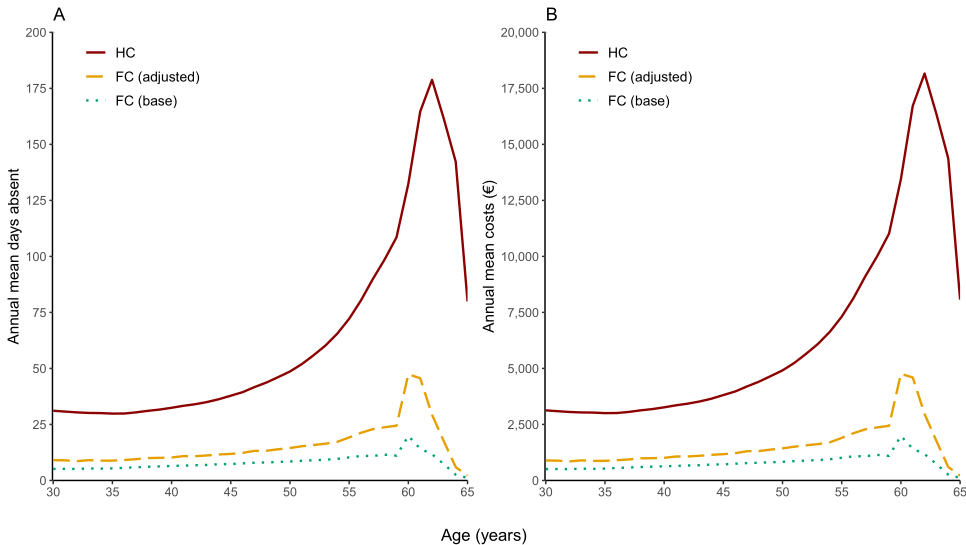


**ESM Figure 2.** Friction cost estimates of people with diabetes (restricted people with known occupation) by by their employment status (A), by highest degree of education (B), by sex (C), and by birth cohort (D). In figure (D), the annual mean cost estimate at age 48 for people born in the 1930s and 1940s is based on 40 observations.


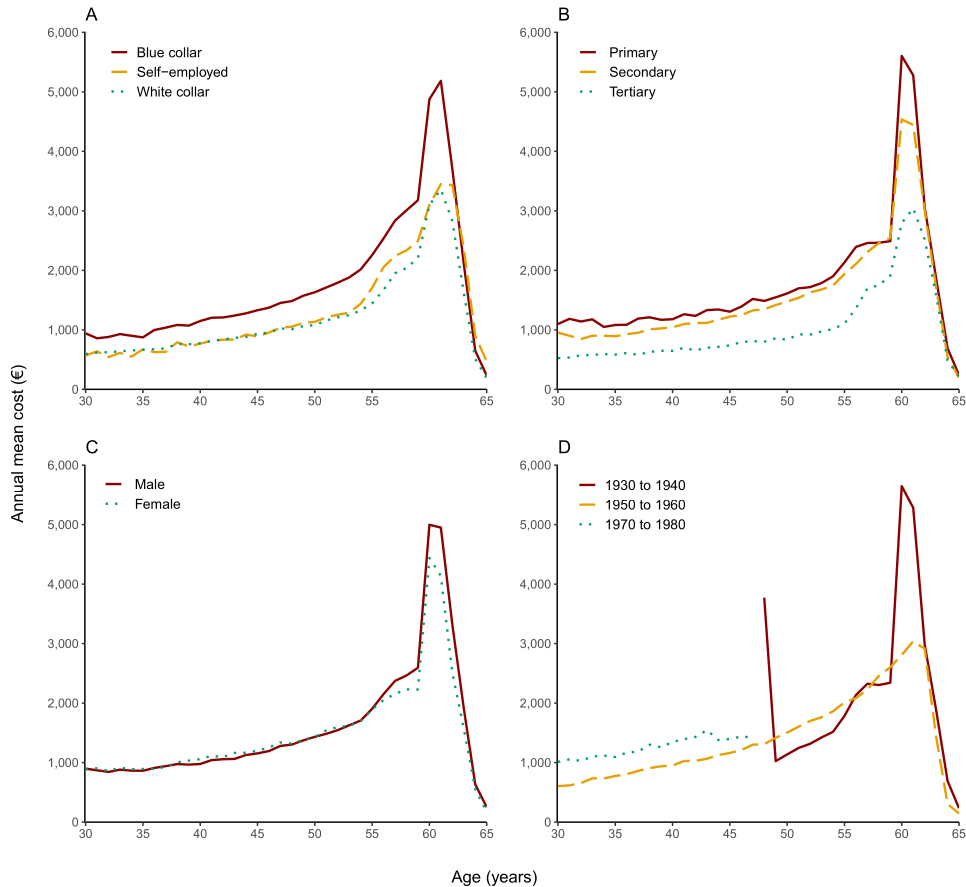

Supplement: Supplementary file 1 — Supplementary Material 1. [file 13561_2026_722_MOESM1_ESM.docx]
